# Brazil at the Forefront: What Does It Take To Be Net‐Zero by 2040

**DOI:** 10.1111/gcb.71090

**Published:** 2026-09-15

**Authors:** Gerd Angelkorte, Nathália Nascimento, Eduardo Delgado Assad, Luiz Bernardo Baptista, Mercedes Bustamante, Sabrina Matos Carlos, Fabio Diuana, Felipe Lenti, Núbia Marques, Susian Martins, Laura Vanini Polli, Melina Sampaio, Roberto Schaeffer, Daiane Pereira de Sousa, Alice Assad Wassal, Carlos A. Nobre

**Affiliations:** ^1^ Centre for Energy and Environmental Economics (Cenergia), Energy Planning Program (PPE), Coppe Universidade Federal do Rio de Janeiro Rio de Janeiro Rio de Janeiro Brazil; ^2^ Departamento de Ciências Florestais Universidade de São Paulo Piracicaba São Paulo Brazil; ^3^ Bioeconomy Observatory Getulio Vargas Foundation FGV/GVagro São Paulo Brazil; ^4^ Department of Ecology, Institute of Biology University of Brasília Brasília Brazil; ^5^ Fauna Projetos Corporation Campinas São Paulo Brazil; ^6^ Institute of Advanced Studies University of São Paulo Butantã Brazil

**Keywords:** AFOLU, bioenergy, energy system decarbonization, integrated assessment model, land‐use mitigation, mitigation pathways, net‐zero pathways, reforestation

## Abstract

Brazil's current Nationally Determined Contribution (NDC) establishes a target of net‐zero greenhouse gas (GHG) emissions by 2050. Here, we use the Brazilian Land Use and Energy Systems model (BLUES) to investigate whether national GHG neutrality could be advanced to 2040. We compare a reference pathway reaching net zero in 2050 with two stylised accelerated pathways: AFOLU‐2040, which prioritises mitigation in agriculture, forestry and other land use, and Energy‐2040, which emphasises energy‐system transformation. All pathways incorporate the same economy‐wide upper‐bound constraints associated with Brazil's 2030 and 2035 NDC commitments. However, because the accelerated pathways combine an earlier neutrality constraint with sector‐specific assumptions, their comparison with Net‐Zero 2050 does not isolate the effect of changing the net‐zero year alone. Both accelerated pathways reach net zero in 2040, but their near‐term trajectories diverge. By 2035, net GHG emissions decline to 850 MtCO_2_e.yr^−1^ in Net‐Zero 2050, 655 MtCO_2_e.yr^−1^ in Energy‐2040 and 627 MtCO_2_e.yr^−1^ in AFOLU‐2040. AFOLU‐2040 relies on rapid deforestation control, ecosystem restoration and changes in agricultural production systems, whereas Energy‐2040 entails earlier transport electrification, renewable‐electricity expansion, advanced biofuels and carbon capture and storage. Relative to Net‐Zero 2050, cumulative territorial GHG emissions are 1.17 GtCO_2_e lower in Energy‐2040 and 1.45 GtCO_2_e lower in AFOLU‐2040 over the modelled period. Regional results further show that land‐use change, electricity expansion and biofuel production are distributed unevenly across Brazil's macro‐regions. Advancing neutrality is therefore technically feasible under the model assumptions, but implementation would require substantially different near‐term policies, infrastructure and governance capacities, along with regionally differentiated planning and coordination across levels of government.

## Graphical abstract

Using the BLUES integrated assessment model, we show Brazil can reach net‐zero emissions a decade early, by 2040, via two contrasting pathways: one prioritising deforestation elimination and low‐carbon agriculture (AFOLU‐2040), the other emphasising transport electrification, renewables and industrial decarbonisation (Energy‐2040) – both requiring earlier near‐term action.

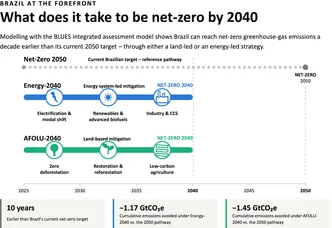

## 1 Introduction

In 2023, the global mean temperature exceeded an average increase of 1.5°C during the period from 2023 to 2025, surpassing the threshold established by the Paris Agreement, which calls on nations to progressively increase their ambitions to limit global warming to 1.5°C (Calvin et al. 2023; IPCC 2018). Brazil, as a major emitter and a country with globally significant ecosystems, has committed to reach greenhouse gas (GHG) net‐zero emissions by 2050, starting with a reduction in GHG emissions of between 59% and 67% by 2035, taking the year 2005 as a baseline (Brasil 2024a). This pledge is already ambitious, as it aims to achieve net‐zero CO_2_ emissions by 2040 (Brasil 2024a). Given its unique position, with vast forests, substantial areas of degradation, a strong agricultural sector, an advanced bioenergy industry, and a diverse economy, we test here the hypothesis of whether or not Brazil may be able to accelerate this pathway.

In 2024, during the 3rd Session of the G20 Leaders' Meeting in Rio de Janeiro, President Luiz Inácio Lula da Silva called upon all nations to strengthen their climate commitments and to bring forward their net‐zero GHG pledges by 5–10 years, encouraging targets of 2040–2045 rather than 2050 (Brasil 2024a, 2024b, 2024c). This call reinforces Brazil's leadership role in global climate politics: a country that already holds an ambitious domestic pledge, but that also urges the international community to move faster. Against this backdrop, the originality of our study lies in testing what it would mean for a large emerging economy such as Brazil to advance its own net‐zero target by a decade and in evaluating the broader implications of such a move across key sectors, for development, governance, and diplomacy. By doing so, we also aim to provide lessons that may inspire other nations to follow a similar path.

Previous studies have examined Brazil's long‐term mitigation pathways using national integrated assessment models, sectoral models and economy‐wide analyses. La Rovere et al. (La Rovere et al. 2018) investigated the economic, social and financing implications of deep decarbonisation in Brazil, highlighting the importance of coordinating climate policy with development and investment strategies. Using the BLUES model, Köberle et al. (Köberle et al. 2020) showed that stringent mitigation pathways require a combination of avoided deforestation, agricultural intensification, transport electrification, advanced biofuels and carbon capture and storage. More recently, Soterroni et al. (Soterroni et al. 2023) demonstrated that eliminating legal and illegal deforestation and expanding ecosystem restoration could bring Brazil close to net‐zero emissions around 2040, while Poggio et al. (Poggio et al. 2024) highlighted the importance of biofuels, bioenergy with carbon capture and storage and the interaction between domestic least‐cost choices and global technological trends. Collectively, these studies establish the central role of AFOLU mitigation and energy‐system transformation in Brazil. To our knowledge, these studies do not explicitly compare the current 2050 net‐zero GHG target with an accelerated 2040 target under Brazil's latest 2030 and 2035 NDC constraints, while distinguishing between stylised land‐led and energy‐led mitigation portfolios. This comparison constitutes the principal contribution of the present study.

To address this gap, we use the Brazilian Land Use and Energy Systems model (BLUES), a national integrated assessment model (IAM) (Angelkorte 2023; Rochedo et al. 2018; Angelkorte and Rochedo 2026), to compare three pathways: a reference pathway reaching net‐zero GHG emissions in 2050 and two accelerated variants reaching net zero in 2040. AFOLU‐2040 emphasizes land‐based mitigation, whereas Energy‐2040 emphasizes energy‐system transformation. Our analysis examines how these contrasting designs affect near‐term emissions, land‐use change, carbon‐removal portfolios, primary energy, electricity generation, and transport electrification. The scenarios are intended as stylised mitigation strategies rather than forecasts of Brazil's most likely technological development.

## 2 Methods

### 2.1 Model Framework and Optimisation Structure

We employed the BLUES model, an IAM developed for the Brazilian context. The BLUES model is a least‐cost optimisation and mixed‐integer programming model that assumes long‐term perfect foresight through 2060 with 5 year optimisation timesteps (Rochedo et al. 2018; IPCC 2022a; IAMC 2007). The BLUES model was developed using the International Institute for Applied Systems Analysis (IIASA) MESSAGE (Model for Energy Supply Strategy Alternatives and their General Environmental impacts) platform. It is a bottom‐up model representing all five Brazilian macro‐regions (South, Southeast, Midwest, North, and Northeast) plus the Brazil region, which accounts for all exogenous food demand and commodity imports and exports (Angelkorte 2023; Rochedo et al. 2018; IAMC 2007). The model provides high detail on conventional and new technologies across energy, land‐use, and water‐use systems, considering principal inputs such as investments, operation and maintenance costs, agricultural inputs, and others (Köberle et al. 2020; Angelkorte 2023; Rochedo et al. 2018; Angelkorte and Rochedo 2026; IAMC 2007; de Oliveira et al. 2021; Müller‐Casseres et al. 2021; Diuana 2022). In the energy sector, the model has a level of detail that encompasses, simultaneously, the transformation, transport (passenger and freight), and consumption sectors (Rochedo et al. 2018).

The model is formally calibrated to the Brazilian energy and land‐use systems in 2020. The 2025 timestep represents the first modelled optimisation period; its initial conditions were anchored to the most recent historical observations available when the scenarios were constructed, adjusted by recent trends until 2024. Historical national GHG inventory, deforestation and energy‐balance series therefore informed the model's calibration and initialisation. Scenario results from 2025 onwards should therefore be interpreted as normative, target‐consistent pathways rather than as forecasts of realised emissions or technology deployment. Observations that became available only after the scenarios had been constructed—including Brazil's national GHG inventory through 2022 (Brazil 2024), PRODES deforestation estimates through 2025 (INPE Instituto Nacional de Pesquisas Espaciais 2025) and Brazilian Energy Balance data through 2025 (EPE Empresa de Pesquisa Energética 2026) – are presented only as external contextual benchmarks for recent national trends and were not used for point‐by‐point validation of the BLUES trajectories or imposed as model outcomes beyond the calibration and initialisation period.

### 2.2 Representation of Energy and AFOLU Systems

Currently, in the AFOLU sector, the BLUES model identifies six large groups of agricultural production technologies, encompassing both traditional production systems, high‐productivity systems, organic production, double‐crop systems, integrated systems, and disruptive agricultural technologies (Angelkorte 2023). In addition, the model accounts for the principal chemical and organic inputs for crop production under each agricultural production technology, such as fertilisers, pesticides, and irrigation water (Angelkorte 2023; Rochedo et al. 2018; IAMC 2007). Moreover, it considers the costs associated with these productions, the GHG emissions generated, the level of mechanisation required, the amount of diesel used, and the expansion potential of each production system (Angelkorte 2023; Rochedo et al. 2018; IAMC 2007). Thus, each means of agricultural production is developed individually and regionalised for each of these main inputs (Rochedo et al. 2018; IAMC 2007).

Furthermore, the model has ten land‐cover classes forest, savanna, degraded pasture, non‐degraded pasture, crop, double‐crop, planted forest, integrated crop‐livestock system, integrated crop‐livestock‐forestry with commercial forestry system (ICLFc), and integrated crop‐livestock‐forestry with native forest system (ICLFn) (Angelkorte 2023; Angelkorte and Rochedo 2026).

### 2.3 Scenario Design and Policy Assumptions

For this study, we developed three scenarios that incorporate the same economy‐wide emissions constraints associated with Brazil's 2030 and 2035 NDC commitments. Net‐Zero 2050 represents Brazil's current net‐zero GHG target, whereas AFOLU‐2040 and Energy‐2040 bring the neutrality year forward by a decade and explore contrasting sectoral mitigation strategies. So, for that, we developed: (1) Brazil's Baseline NDC trajectory (Net‐Zero 2050): (i) Scenario based on the current Brazilian net‐zero GHG target (Brasil 2024a); (2) AFOLU‐2040 scenario (AFOLU‐2040): (i) Strong reduction in deforestation in native areas in the short term, ending the illegal and legal suppression of native vegetation until 2030; (ii) Expansion of reforestation and restoration programs, even greater than the restoration agreements already established nationally by the National Plan for Native Vegetation Recovery (Planaveg) (12 Mha until 2030); (iii) Adoption of low‐carbon agricultural practices, exceeding the ABC+ plan (Brazil's national policy for fostering climate‐smart agriculture) goals; (iv) Emissions reductions primarily from land‐use change and agriculture, considering other practices not already described, like enzyme utilisation, alternative proteins and others; and (3) Energy‐2040 scenario (Energy‐2040): (i) Rapid decline in oil demand driven by electrification and modal shifts; (ii) Reduced reliance on exporting oil and its derivatives; (iii) Large‐scale deployment of Carbon Capture and Storage (CCS) technologies; (iv) Expansion of advanced biofuels and renewable power; (v) Decarbonization of industry and transport.

All three scenarios include the same economy‐wide emissions constraints associated with Brazil's 2030 and 2035 NDC commitments. These interim targets are implemented as upper bounds on net national GHG emissions rather than as fixed emissions outcomes. The scenarios can therefore overachieve the NDC targets and follow different sectoral trajectories. Because BLUES optimises investments and technology deployment intertemporally under perfect foresight, the terminal net‐zero constraint influences mitigation decisions in the preceding periods.

The scenario ensemble was designed to explore stylised sectoral strategies to reach net‐zero in 2040 rather than to isolate the causal effect of changing the net‐zero year. Net‐Zero 2050 does not impose an additional ex ante preference for either AFOLU‐ or energy‐sector mitigation beyond the common policy assumptions. AFOLU‐2040 and Energy‐2040 combine an earlier net‐zero constraint with scenario‐specific assumptions that favour different mitigation portfolios. Consequently, the differences observed in 2030 and 2035 reflect the combined effects of the earlier neutrality constraint and the sector‐specific scenario design. Comparisons between Net‐Zero 2050 and either accelerated scenario should therefore not be interpreted as representing the isolated effect of advancing net zero from 2050 to 2040.

All scenarios use the same socioeconomic drivers, sectoral demands, resource potentials and techno‐economic assumptions unless otherwise stated. The limited set of scenario‐specific constraints and adjustments is summarised in Table 1. Quantitative changes in land allocation, energy supply, emissions and technology deployment are endogenous optimisation results rather than exogenously prescribed outcomes, unless explicitly identified as constraints in the table.

**TABLE 1:** Summary of common and scenario‐specific assumptions used in the three net‐zero pathways.

Scenario Dimension	Net‐Zero 2050	AFOLU‐2040	Energy‐2040
Main scenario objective	Sector‐neutral least‐cost mitigation	AFOLU‐led mitigation	Energy‐system‐led mitigation
NDC targets	Incorporates Brazil's 2030 and 2035 NDC commitments	Incorporates Brazil's 2030 and 2035 NDC commitments; overachievement is allowed	Incorporates Brazil's 2030 and 2035 NDC commitments; overachievement is allowed
Net‐zero GHG	Net‐zero GHG emissions in 2050	Net‐zero GHG emissions in 2040	Net‐zero GHG emissions in 2040
Deforestation	No additional scenario‐specific acceleration beyond the common policy assumptions	Stronger near‐term reduction in native‐vegetation loss, including elimination of legal and illegal deforestation by 2030	No additional AFOLU‐specific strengthening beyond the common assumptions
Restoration and reforestation	Deployment determined endogenously under the 2050 target and existing restoration commitments	Accelerated restoration and reforestation beyond existing national commitments, including Planaveg (12 Mha)	Restoration and reforestation levels under existing national commitments reduced, reaching a maximum of 50% of Planaveg targets
Low‐carbon agriculture	Adoption is determined endogenously under the common technology and policy assumptions, respecting the objectives of the ABC+ Plan	Accelerated adoption of low‐carbon agricultural practices beyond the ABC+ targets	10‐year delay in the implementation of the agricultural and livestock objectives of the ABC+ Plan
Transport electrification and modal shifts	Deployment determined endogenously under the 2050 constraint	No additional scenario‐specific acceleration	Increased permission for transport electrification and modal shifts
Oil consumption and refining	Oil consumption and refinery activity determined endogenously under the 2050 target	No additional scenario‐specific restriction on progressive oil reduction; enforcing minimum refinery utilization to maintain a minimum utilization level of 70%	Endogenous model choice, without forcing minimum refinery utilisation
Renewable electricity	Expansion determined endogenously	Expansion determined endogenously	Expansion determined endogenously
Advanced biofuels	Deployment determined endogenously	Deployment determined endogenously	Deployment determined endogenously
CCS	Deployment determined endogenously under the 2050 constraint	No additional preference; mitigation is predominantly land‐based	Greater availability and deployment of CCS
Industrial decarbonisation	Determined endogenously under the 2050 target	No additional scenario‐specific acceleration	Accelerated fuel substitution and industrial decarbonisation
Interpretation	Reference pathway consistent with the current 2050 target	Stylised land‐based mitigation stress test	Stylised energy‐system mitigation stress test

*Note:* ‘Endogenous’ indicates that the corresponding level is selected by the cost‐optimization model rather than prescribed exogenously. Unless explicitly stated, all socioeconomic, demand, resource and techno‐economic assumptions are identical across scenarios.

Abbreviations: ABC+, Brazilian plan for adaptation and low‐carbon emissions in agriculture; AFOLU, agriculture, forestry and other land use; CCS, carbon capture and storage; GHG, greenhouse gas; NDC, nationally determined contribution; Planaveg, National Plan for Native Vegetation Recovery.

Detailed common model settings and the quantitative implementation of the scenario‐specific constraints are reported in Table S1. Selected modelled outcomes are reported in Table S2.

Unless otherwise stated, the scenarios share the same socioeconomic drivers, sectoral demands, resource potentials and techno‐economic assumptions. The numerical constraints and implementation periods that differ among the pathways are documented in Table S1.

## 3 Results

### 3.1 Alternative Pathways to Net‐Zero by 2040

Brazil's latest official national inventory reports net GHG emissions of approximately 2039 MtCO_2_e in 2022. The pathways presented below begin in 2025 and represent modelled outcomes consistent with Brazil's climate targets rather than short‐term forecasts. The difference between the latest inventory estimate and the modelled 2025 timestep illustrates the substantial near‐term mitigation effort embedded in the NDC‐consistent pathways.

**FIGURE 1:**
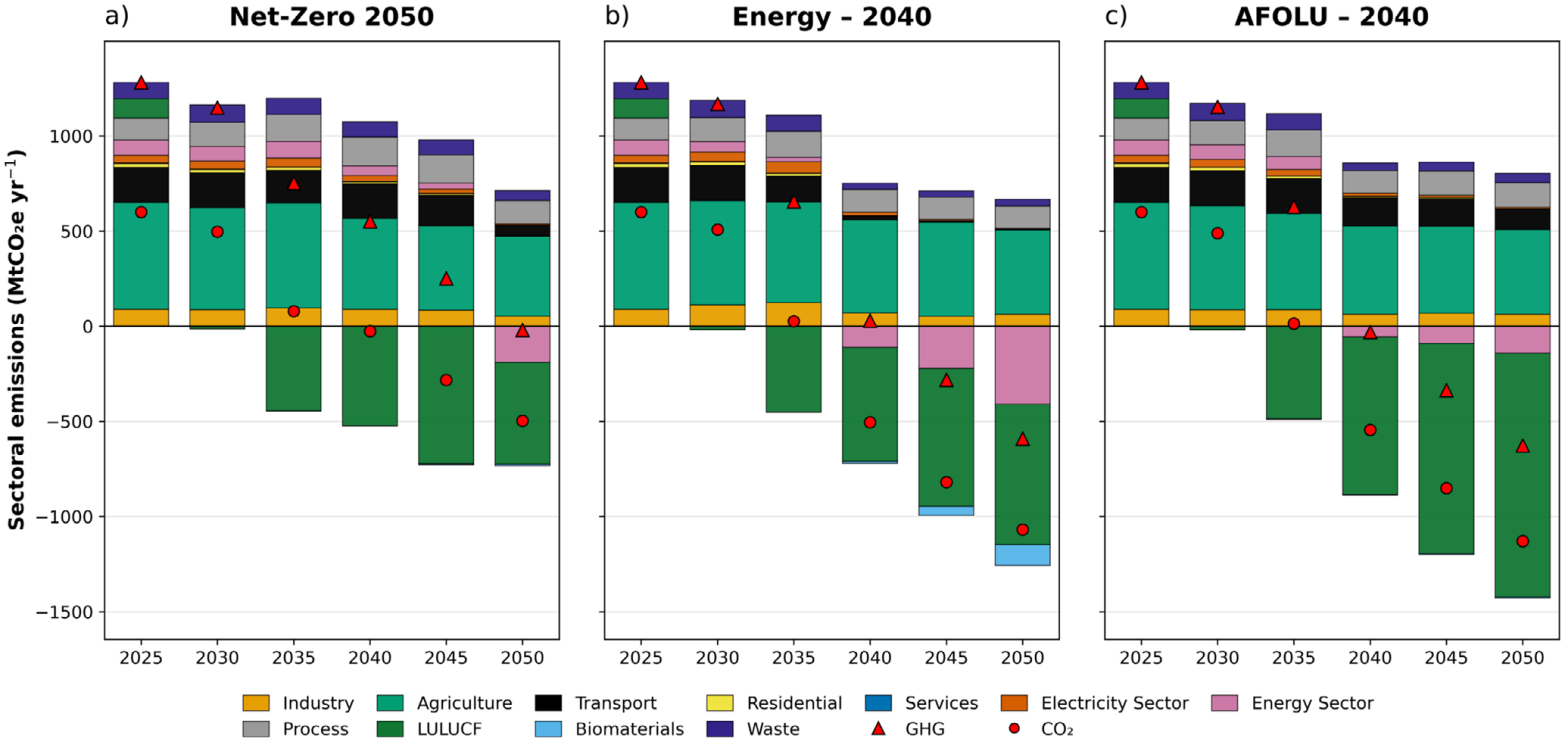
National GHG emission trajectories under alternative pathways. Sectoral emissions under: (a) Net‐Zero 2050; (b) Energy‐2040; and (c) AFOLU‐2040. All values shown correspond to BLUES model results, with 2025 representing the first modelled timestep. For context, Brazil's NDC establishes economy‐wide net GHG emissions targets of 1200 MtCO2e.yr‐1 in 2030 and 850–1050 MtCO2e.yr‐1 in 2035. These interim commitments are considered in all three scenarios. Net‐Zero 2050 reflects Brazil's current neutrality target and reaches net‐zero GHG emissions by mid‐century. The two accelerated scenarios explore alternative strategies for bringing neutrality forward to 2040: AFOLU‐2040, in which reductions are driven primarily by avoided deforestation, restoration and reforestation; and Energy‐2040, in which fossil‐fuel demand declines, bioenergy expands and CCS deployment increases. Red triangles represent net national GHG emissions, while red circles represent net national CO_2_ emissions. Net GHG emissions reach zero in 2050 in panel a and in 2040 in panels b and c. GHG, Greenhouse gases; LULUCF, Land‐use change and forestry.

Brazil's baseline NDC trajectory achieves net‐zero GHG emissions by 2050, consistent with the country's official pledge under the Paris Agreement. This trajectory reflects incremental progress in reducing emissions across all major sectors, with land‐use policies gradually tightening and energy transitions occurring at a pace consistent with global mid‐century neutrality targets. The other two scenarios, AFOLU‐2040 and Energy‐2040, were supported by results from BLUES, which confirmed that Brazil has the technical capacity to achieve net‐zero emissions a full decade earlier, reaching that by 2040 through alternative sectoral strategies. These accelerated pathways, AFOLU‐2040 and Energy‐2040, rely on different combinations of mitigation measures, with the former driven primarily by land‐use change interventions and the latter by structural transformations in the energy system. Both converge to net zero emissions by 2040, but their temporal dynamics, sectoral contributions, and technology portfolios differ substantially (Figure 1).

Near‐term emissions remain relatively similar across the three pathways in 2030, when net national GHG emissions reach approximately 1200, 1168 and 1153 MtCO_2_e.yr^−1^ in Net‐Zero 2050, Energy‐2040 and AFOLU‐2040, respectively. The two more restrictive scenarios present values below Brazil's 2030 NDC target of 1200 MtCO_2_e.yr^−1^. The divergence becomes more pronounced by 2035, when emissions decline to approximately 850, 655 and 627 MtCO_2_e.yr^−1^, respectively. Thus, all more restrictive scenario pathways overachieve even the more ambitious end of Brazil's 2035 NDC range of 850–1050 MtCO_2_e.yr^−1^. These results indicate that advancing neutrality not only changes the final year of the pathway but also affects the scale, timing and sectoral composition of mitigation during 2025–2035. AFOLU‐2040 front‐loads reductions through deforestation control, ecosystem restoration and changes in agricultural production systems, whereas Energy‐2040 requires earlier investments in transport electrification, renewable electricity, advanced biofuels and industrial decarbonisation. Over the modelled period, cumulative net national GHG emissions are 1.17 GtCO_2_e lower in Energy‐2040 and 1.45 GtCO_2_e lower in AFOLU‐2040 than in Net‐Zero 2050. These values represent differences in cumulative territorial emissions within Brazil and should not be interpreted as globally avoided emissions.

In the AFOLU‐2040 scenario, emissions decline steeply from the mid‐2020s as deforestation is curtailed at least by 50% relative to 2020 levels within a decade (in all biomes), and large‐scale reforestation and restoration programs begin to store carbon at increasing rates. By the early 2030s, net GHG emissions from AFOLU approach zero. By 2040, net AFOLU emissions reach approximately −370 MtCO_2_e.yr^−1^, resulting from approximately 460 MtCO_2_e.yr^−1^ of agricultural emissions and approximately 830 MtCO_2_e.yr^−1^ of removals from land‐cover change. Net AFOLU emissions become increasingly negative thereafter, reaching approximately −1.1 GtCO_2_e.yr^−1^ in 2050. This occurs because of the continued sequestration of forest carbon from reforestation and restoration measures foreseen in the AFOLU‐2040 scenario, and from the reduction in the agricultural sector's equivalent carbon footprint, primarily methane.

This pathway delivers immediate reductions and positions the land sector as the dominant contributor to early neutrality. By contrast, the Energy‐2040 pathway reduces emissions more gradually in the early years but accelerates rapidly after 2030. Fossil fuel demand falls sharply, oil use declines by 55% by 2035, refinery output contracts by 41%, and exports fall by 71% compared to the Net‐Zero 2050 scenario. The electrification of transport reinforces these changes, reaching, by 2040, an electrification corresponding to 31% (7% in the AFOLU‐2040 scenario) of the fleet's energy consumption, with 49% (8% in the AFOLU‐2040 scenario) of the passenger vehicle fleet's energy consumption being done through electricity and 8% (6% in the AFOLU‐2040 scenario) of the freight vehicle fleet's energy consumption. Furthermore, the replacement of industrial fuels, the expansion of renewable energy, and the widespread implementation of CCS stand out as fundamental. Together, these measures drive a structural transformation in the Brazilian economy, making the energy sector the main driver of decarbonisation.

### 3.2 Divergent Land‐Use Transformation Dynamics

The modelled land‐use pathways occur against a background of recent reductions in deforestation, although absolute forest losses remain substantial. PRODES recorded 5796 km^2^ of deforestation in the Brazilian Amazon and 7235 km^2^ in the Cerrado in 2025, representing reductions of 11.08% and 11.49% relative to 2024, respectively (INPE Instituto Nacional de Pesquisas Espaciais 2025). These recent declines demonstrate that rapid improvements in forest governance are possible, but the remaining deforestation levels also indicate the additional effort required to approach the near‐zero deforestation trajectory represented in AFOLU‐2040.

**FIGURE 2:**
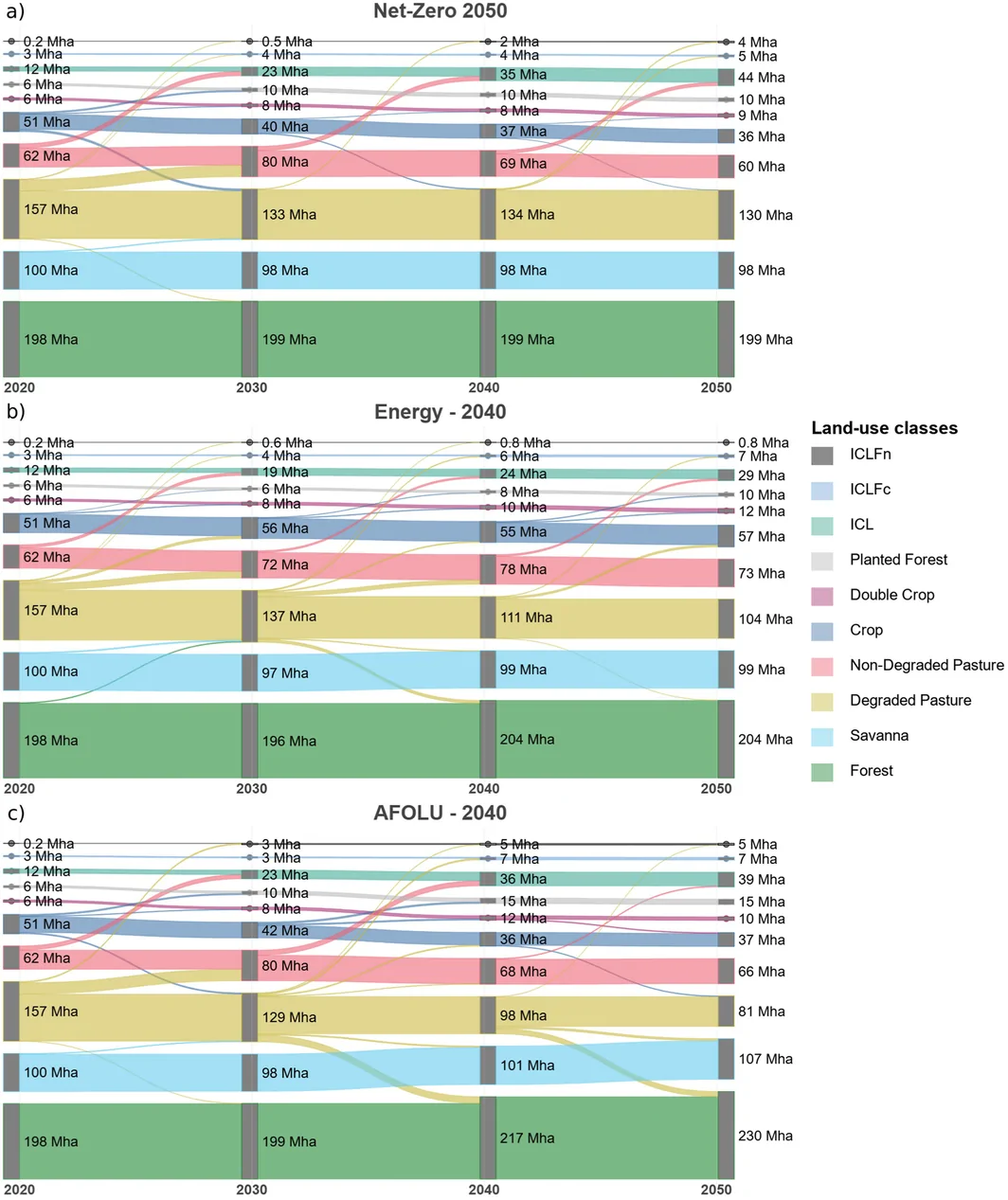
Projected land‐use allocation under alternative net‐zero pathways. Land‐use allocation between 2020 and 2050 under (a) Net‐Zero 2050, (b) Energy‐2040 and (c) AFOLU‐2040. Values are expressed in millions of hectares (Mha). The panels use the same category order and colour scheme to facilitate comparison across scenarios. ICL, Integrated crop‐livestock; ICLFc, Commercial integrated crop‐livestock‐forest; ICLFn, Native integrated crop‐livestock‐forest; Non‐Degraded Pasture: Pasture that refers to pasture without the degradation characteristics represented in the degraded‐pasture class.

**FIGURE 3:**
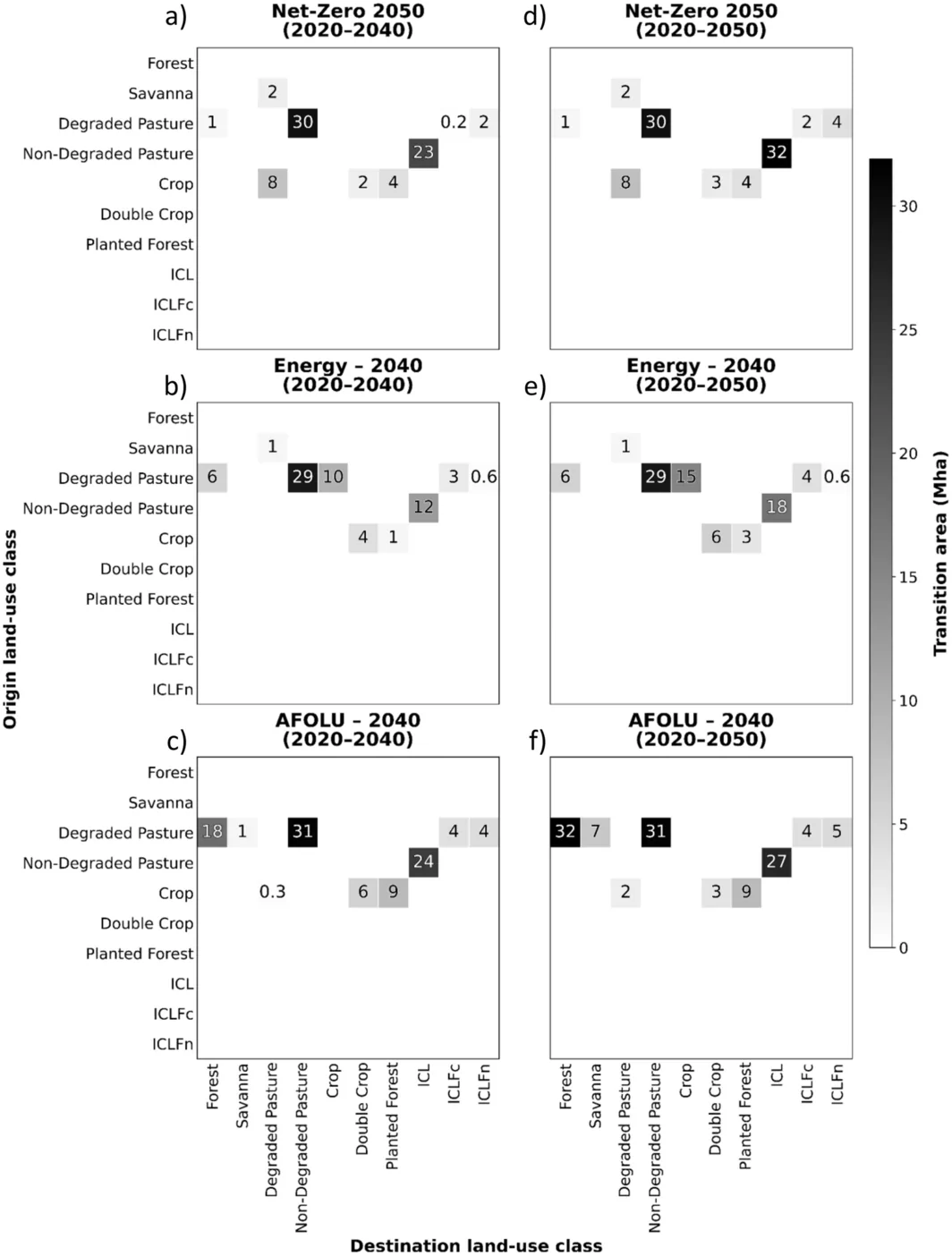
Cumulative land‐use flows under alternative net‐zero pathways. Panels (a–c) show cumulative land‐use flows between 2020 and 2040 under Net‐Zero 2050, Energy‐2040, and AFOLU‐2040, respectively. Panels (d–f) show the corresponding cumulative flows between 2020 and 2050. The Y‐axis identifies the initial land‐cover class, and the X‐axis identifies the final land‐cover class. Values represent the area transferred between classes and are expressed in millions of hectares (Mha). Category order and colours are consistent across all panels. Main‐diagonal values, representing land remaining within the same land‐use class, are omitted; ICL, Integrated crop‐livestock system; ICLFc, Integrated crop‐livestock‐commercial forestry system; ICLFn, Integrated crop‐livestock‐native forestry system; Mha, million hectares; Non‐degraded: Pastrure that refers to pasture without the degradation characteristics represented in the degraded‐pasture class.

Land‐use dynamics emerge as one of the most distinctive features distinguishing pathways toward net‐zero GHG emissions. Figure 2a–c shows the broad reallocation of land across major categories between 2020 and 2050, while Figure 3a–f provides a more granular view of flows between systems at key milestones in 2040 and 2050. Together, these visuals highlight not only the aggregate stock changes but also the direction and scale of transitions required in each scenario.

In the Net‐Zero 2050 baseline (Figures 2a, 3a,d), structural change in land use occurs only gradually. Native forest area increases modestly (1 Mha) over the next two decades, while large tracts (134 Mha) of degraded pasture persist well into the 2040s. By 2040, only small‐scale shifts from degraded to more productive pastures are visible, and reforestation remains limited. Only after 2045 does restoration accelerate, with cumulative transitions from degraded lands to native forest exceeding 30 Mha by 2050. This delayed action reflects the postponement of ambitious land‐use policies under the baseline, consistent with a neutrality target only in mid‐century.

The accelerated scenarios demonstrate much earlier and more pronounced transformations. In the Energy‐2040 pathway (Figures 2b, 3b,e), the dominant feature is a structural intensification of pasture systems. Degraded lands are progressively converted into high‐yield pastures and integrated crop‐livestock‐forestry (ICLF) systems, with over 20 Mha of land shifted to more productive uses by 2040. This reflects a strategy centred on improving efficiency rather than expanding forest cover. Although some restoration occurs, particularly after 2035, the overall expansion of native forests is modest compared to AFOLU‐2040 (Figures 2c, 3c,f). By 2050, degraded pasture is substantially reduced, but net forest gains remain secondary to improvements in agricultural productivity.

**FIGURE 4:**
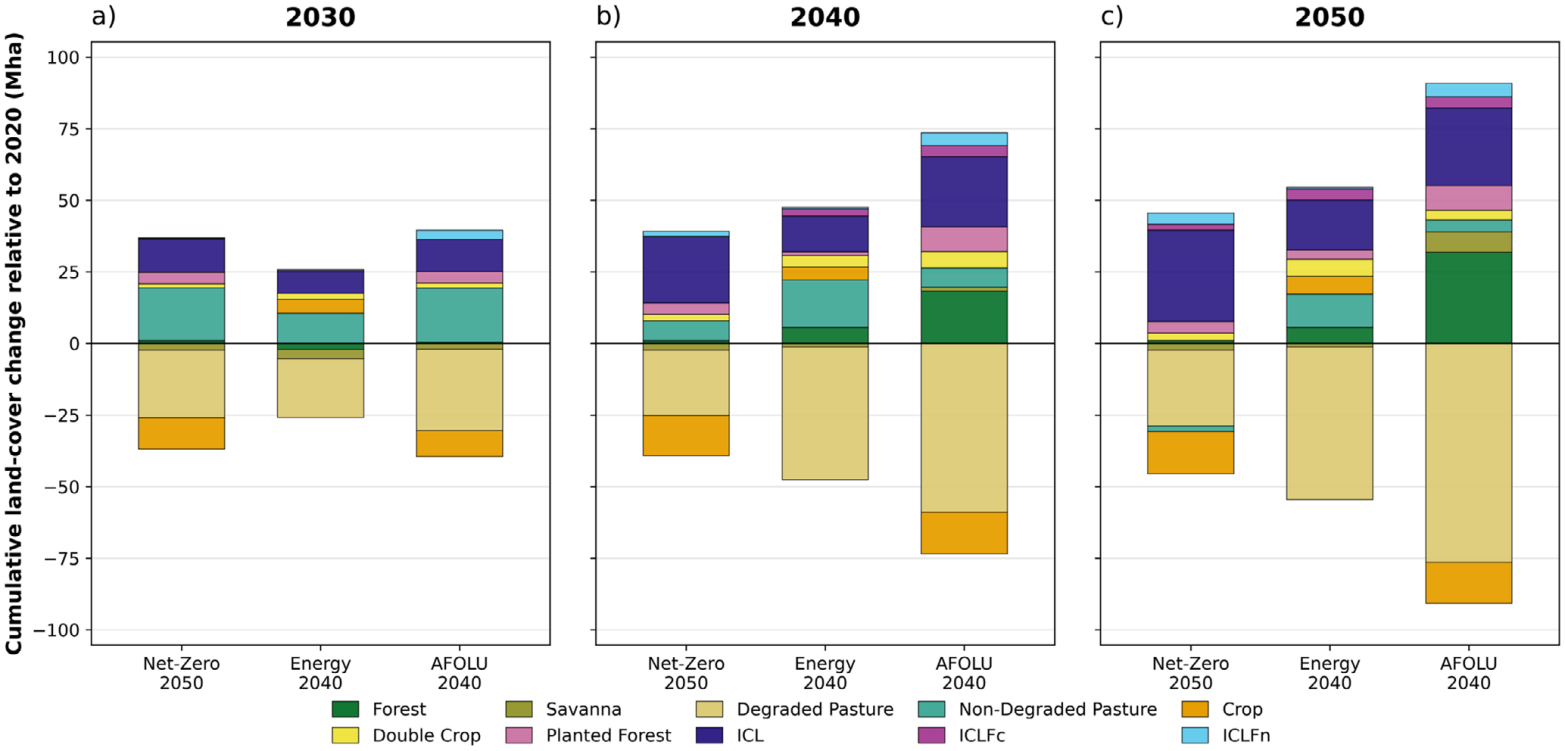
Cumulative land‐use change, relative to 2020, under alternative net‐zero pathways. Cumulative changes in land‐cover area between 2020 and (a) 2030, (b) 2040 and (c) 2050. Within each panel, bars correspond to Net‐Zero 2050, Energy‐2040 and AFOLU‐2040. Positive values indicate an expansion of the corresponding land‐cover class relative to 2020, whereas negative values indicate a contraction. Values are expressed in millions of hectares (Mha). ICL, Integrated crop‐livestock; ICLFc, Commercial integrated crop‐livestock‐forest; ICLFn, Native integrated crop‐livestock; Non‐Degraded Pasture: Pasture without the degradation characteristics.

A very different profile characterises the AFOLU‐2040 scenario (Figures 2c, 3c,f). Here, in the early 2030s, deforestation almost entirely halts, and aggressive reforestation and restoration programs rapidly scale up. By 2040, approximately 20 Mha of degraded pasture will be converted back into native vegetation, with additional transitions toward agroforestry and ICLF systems. Even so, approximately 98 million hectares of degraded pastureland will remain by the end of 2040 in the AFOLU‐2040 scenario. The magnitude of these flows dwarfs the land‐use transitions in the other pathways, underscoring the central role of land‐based mitigation in enabling early neutrality. By 2050, the AFOLU‐2040 scenario achieves the highest net gain in native vegetation area, coupled with a steep reduction in degraded pasture and substantial adoption of integrated systems (Figure 4).

A key insight from the scenario comparison is the temporal distribution of land‐use transformation. In the Net‐Zero 2050 baseline, change is postponed until after 2045, reflecting a strategy of delayed mitigation. In the Energy‐2040 pathway, agricultural intensification is frontloaded, but forest restoration remains comparatively modest until after neutrality is achieved. In AFOLU‐2040, by contrast, transformative land‐use shifts occur early and at scale, with deforestation control and restoration measures operating as the backbone of the mitigation strategy.

These results illustrate two different logics of transition. The Energy‐2040 pathway relies on intensifying production systems to free up land and reduce pressure on forests. At the same time, AFOLU‐2040 relies on large‐scale restoration and avoided deforestation to offset ongoing emissions from fossil fuels. Both strategies deliver net zero by 2040, but through fundamentally different trajectories of land‐use transformation.

### 3.3 Shifting Carbon Dioxide Removal Portfolios

**FIGURE 5:**
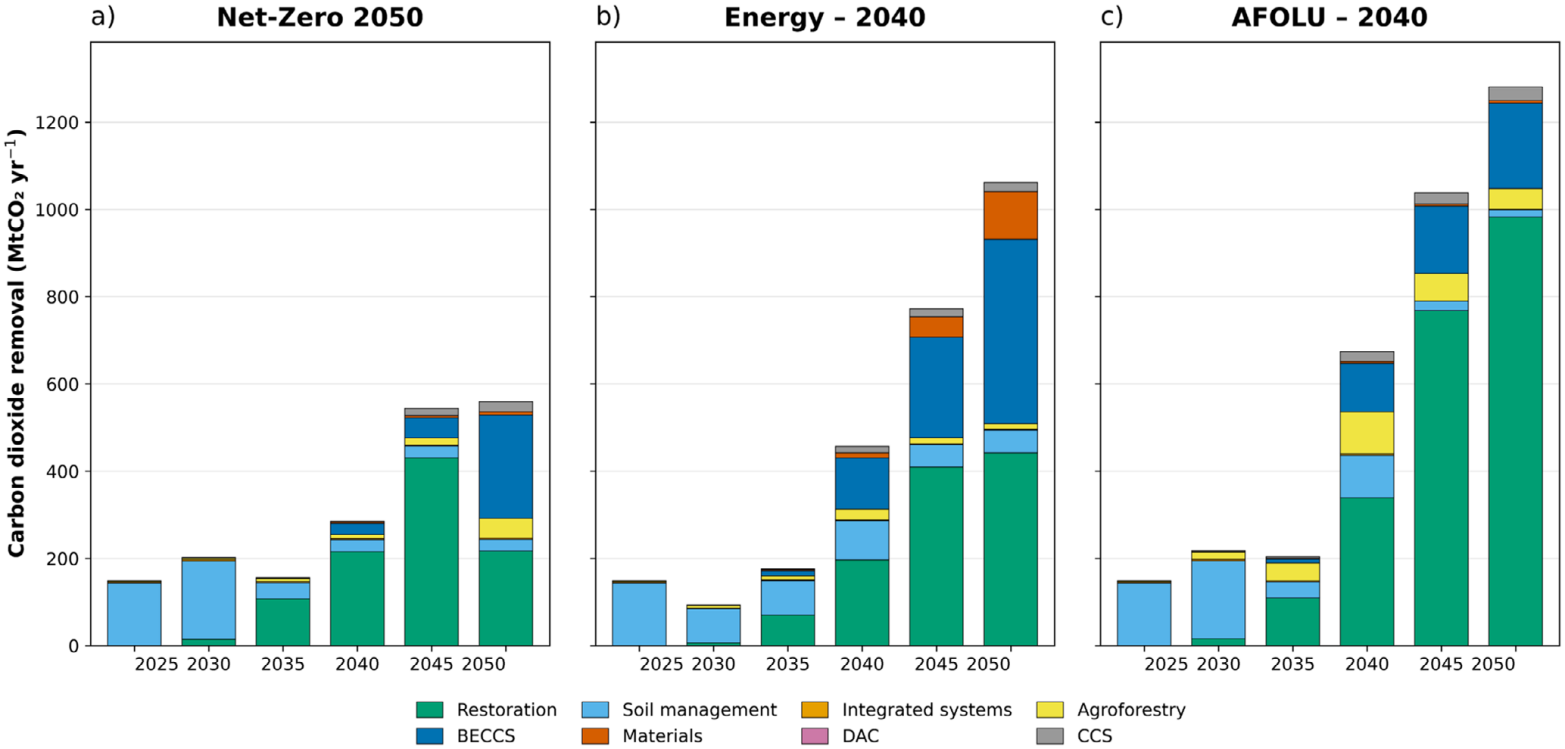
Deployment of carbon dioxide removal (CDR) technologies and fossil and industrial CCS under alternative net‐zero pathways. (a) CDR and CCS technologies from the Net‐Zero 2050 scenario. (b) CDR and CCS technologies from the Energy‐2040 scenario. (c) CDR and CCS technologies from the AFOLU‐2040 scenario. CDR deployment grows substantially after 2030 in all scenarios, but with distinct profiles. AFOLU‐2040 relies primarily on restoration, agroforestry, and integrated land systems, consistent with land‐based mitigation. Energy‐2040 requires greater reliance on BECCS, industrial CCS, and CO_2_ utilisation and storage in materials to offset residual emissions from hard‐to‐abate sectors. Net‐Zero 2050 postpones large‐scale deployment until after 2040, increasing long‐term dependence on technological removals. BECCS, Bioenergy with carbon capture and storage; CCS, carbon capture and storage; CDR, carbon dioxide removal; DAC, direct air capture; Materials: Represents CO_2_ utilization and storage in long‐lived products through CCU/CCUS routes.

Anticipating neutrality also changes the scale, type, and timing of carbon dioxide removal (CDR). In AFOLU‐2040, CDR is dominated by nature‐based solutions, with reforestation, restoration, and agroforestry providing over 87% of total removals by 2040. These measures dominate the optimized land‐based carbon‐removal portfolio and can provide important biodiversity co‐benefits. Still, they depend on strong governance to prevent reversal through illegal deforestation or fire and promote the restoration and adoption of agroforestry systems. In Energy‐2040, the balance shifts toward engineered carbon management. By 2040, BECCS reaches 4 GW of installed capacity and delivers 118 MtCO_2_.yr.^−1^ of carbon removals. Industrial CCS captures an additional 15 MtCO_2_.yr.^−1^ from cement, iron and steel, and chemical production, while material‐based CCU/CCUS pathways, including biopolymers, account for a further 12 MtCO_2_.yr.^−1^ of carbon utilization and storage. This reliance on engineered removals reduces exposure to governance risks but raises new technological and financial uncertainties. The Net‐Zero 2050 baseline postpones large‐scale removals until after 2040, resulting in cumulative additional reliance of 1.1 GtCO_2_e on technological options by mid‐century (Figure 5). This comparison demonstrates that accelerating neutrality reduces total dependence on engineered CDR while exposing each pathway to different categories of risk.

### 3.4 Structural Transformation of the Energy System

Recent observations provide context for the direction of change in the Brazilian electricity sector. According to the Brazilian Energy Balance, solar generation increased by 24.7% and wind generation by 8.2% between 2024 and 2025, reaching 88.1 and 116.5 TWh, respectively. Together, these sources accounted for 26.4% of national electricity generation in 2025. Over the same period, hydropower generation declined by 4.8%, whereas natural‐gas generation increased by 22.7% (EPE Empresa de Pesquisa Energética 2026). These observations are presented to characterise the ongoing electricity supply changes in Brazil. They are not used as a direct calibration or point‐by‐point validation of the BLUES projections, for which 2025 represents the first modelled period.

**FIGURE 6:**
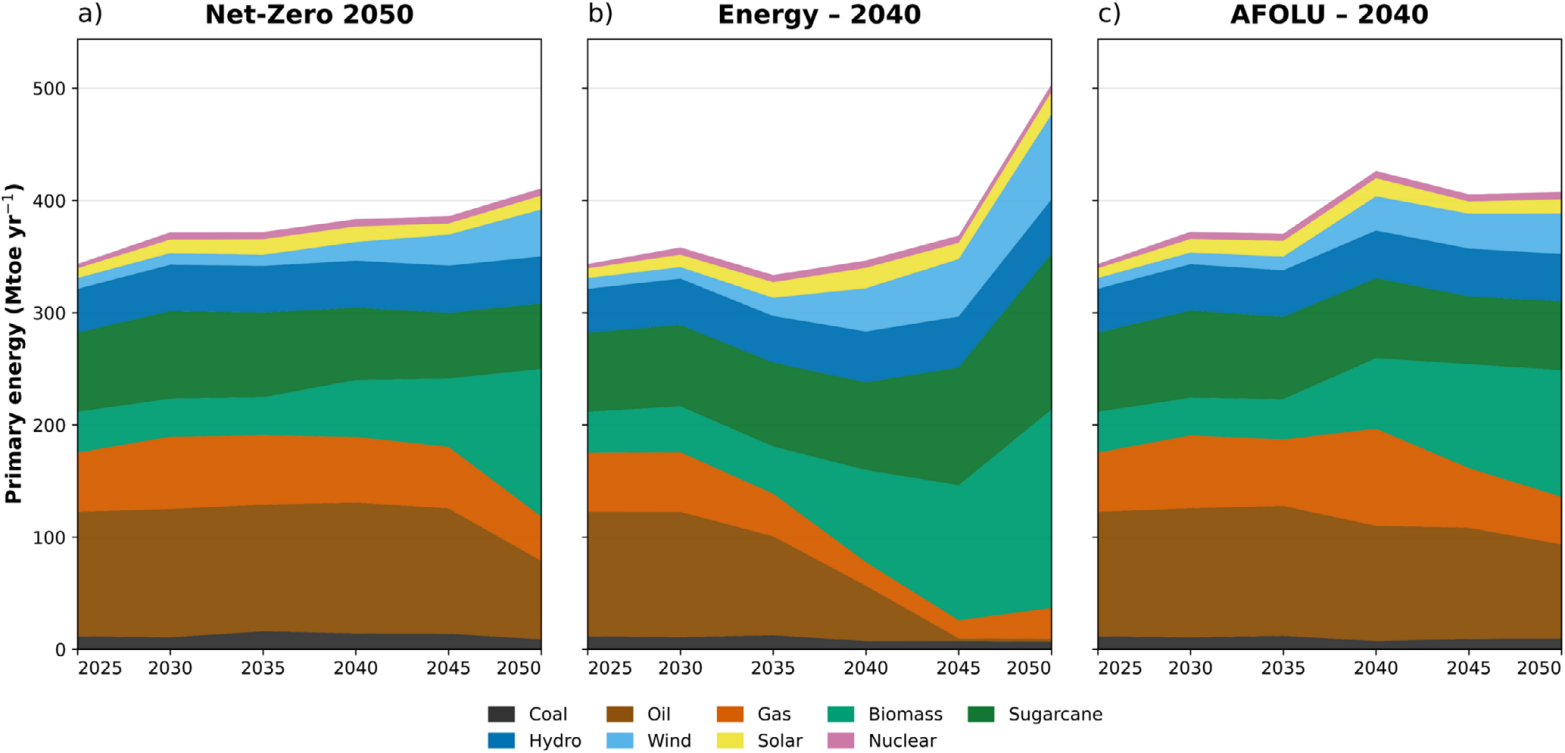
*Primary energy supply across* alternative net‐zero pathways. (a) Primary energy supply from the Net‐Zero 2050 scenario. (b) Primary energy supply from the Energy‐2040 scenario. (c) Primary energy supply from the AFOLU‐2040 scenario. The Energy‐2040 pathway features a steep decline in oil and coal use by the 2030s, offset by a rapid expansion of bioenergy, solar, and wind power, while hydro remains stable. AFOLU‐2040 maintains a more gradual energy transition, with fossil fuels persisting longer, given the dominance of land‐use measures in mitigation. Net‐Zero 2050 delays significant structural changes until after 2040, resulting in higher cumulative emissions. These contrasting pathways reveal alternative strategies for energy transformation, with implications for innovation, security, and the speed of Brazil's decarbonisation.

The transformation of Brazil's energy mix is particularly striking in Energy‐2040 (Figure 6). Fossil fuel use contracts rapidly: oil demand falls by 55% between 2025 and 2040, coal is virtually eliminated by 2035, and natural gas peaks in the early 2030s before entering structural decline. Renewable energy is expanding rapidly, with solar and wind together accounting for 16% of primary energy by 2040, complemented by stable contributions from hydropower and rapid growth in advanced biofuels. By contrast, AFOLU‐2040 maintains a more gradual energy transition, with fossil fuels still accounting for 46% of primary energy in 2040 (in Energy‐2040, this share is only 22%). Still, the sector's emissions are offset by extensive land‐use removals. This slower pace delays the modernization of Brazil's energy and industrial systems and increases the persistence of fossil‐fuel infrastructure.

**FIGURE 7:**
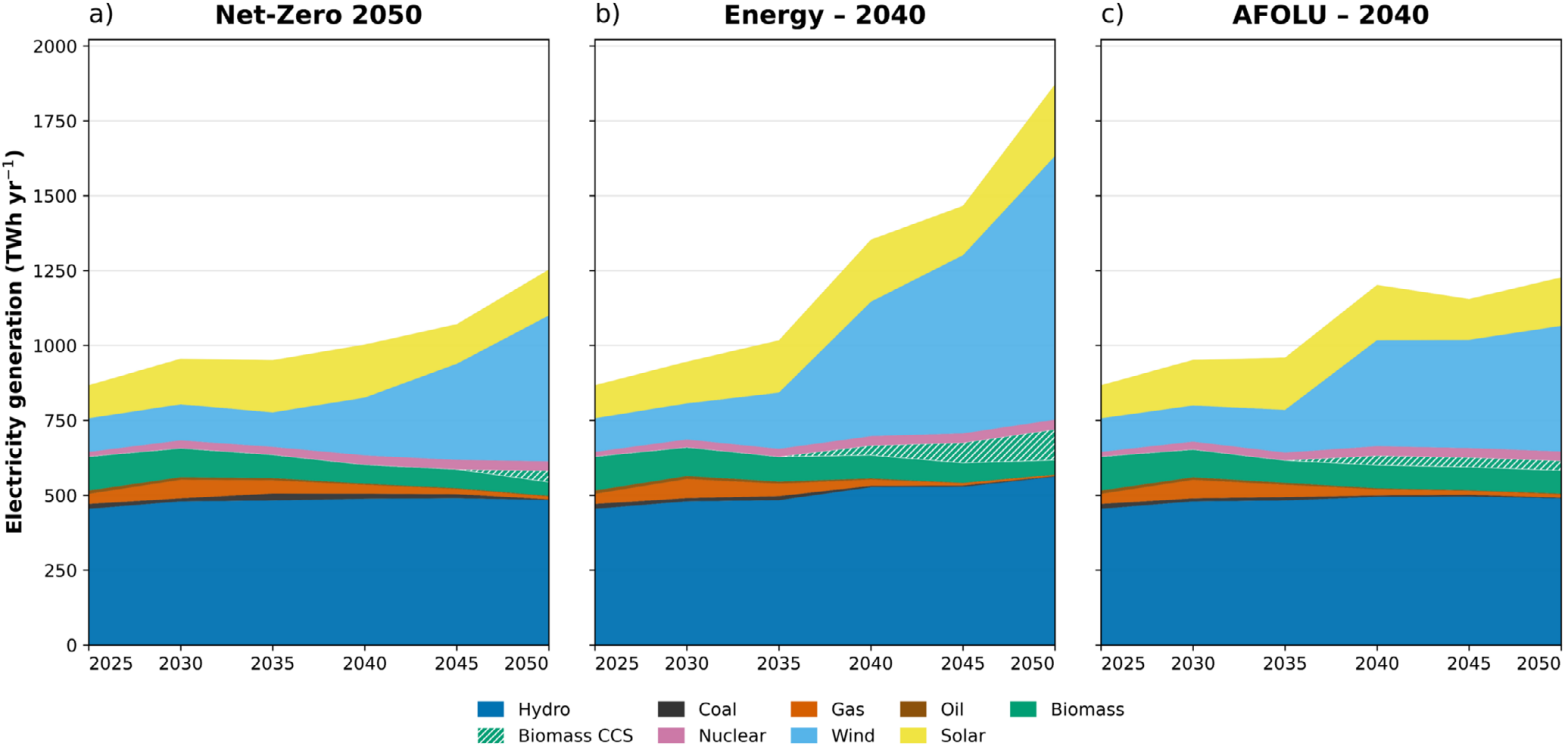
Projected electricity generation under alternative net‐zero pathways. Electricity generation by source under (a) Net‐Zero 2050, (b) Energy‐2040 and (c) AFOLU‐2040. All values are modelled BLUES results, with 2025 representing the first modelled period.

The electricity generation results reveal changes that are less apparent in the primary‐energy representation (Figure 7). Between 2025 and 2035, total electricity generation increases by 9.7% in Net‐Zero 2050, 17.3% in Energy‐2040, and 10.6% in AFOLU‐2040. Hydropower generation increases by approximately 6%, and nuclear generation by approximately 69% across the three pathways. Wind generation remains comparatively stable in Net‐Zero 2050, increasing by 2.5%, but grows by 67.1% in Energy‐2040 and 26.8% in AFOLU‐2040. PV and distributed generation combined increase by approximately 60.6% in all three pathways. Consequently, the combined share of wind, PV, and distributed generation rises from 24.5% in 2025 to 29.1% in Net‐Zero 2050, 34.3% in Energy‐2040, and 31.7% in AFOLU‐2040 by 2035. Thus, the apparent stability of some technologies in Figure 7 partly reflects the use of primary‐energy accounting, which does not fully reveal changes within the electricity‐generation mix.

Transport electrification contributes to the divergence in electricity demand among the pathways. Electricity use in transport increases from 15.6 PJ in 2025 to 112.1 PJ in Net‐Zero 2050, 256.3 PJ in Energy‐2040 and 116.0 PJ in AFOLU‐2040 by 2035. These values correspond to 2.7%, 6.7% and 2.7% of aggregate transport final energy consumption, respectively. By 2040, electricity use reaches 192.8 PJ in Net‐Zero 2050, 700.4 PJ in Energy‐2040 and 286.9 PJ in AFOLU‐2040, corresponding to 4.7%, 31.1% and 7.3% of transport final energy consumption. Relative to 2025, this represents an approximately twelve‐fold increase in Net‐Zero 2050, a forty‐five‐fold increase in Energy‐2040 and an eighteen‐fold increase in AFOLU‐2040. These indicators refer to electricity as a share of aggregate transport final energy consumption and should not be interpreted as shares of vehicle sales or of the vehicle fleet.

### 3.5 Regional Heterogeneity of Mitigation Pathways

National aggregates conceal substantial regional heterogeneity in the deployment of mitigation options (Figures S1–S23). The regional land‐use results show that the AFOLU‐2040 pathway produces particularly pronounced changes in land allocation across the North, Northeast, Midwest, and Southeast, including reductions in degraded pasture and expansion of recovered pasture, native vegetation, and integrated agricultural systems (Figures S4–S8). Agricultural land allocation also differs substantially among pathways, particularly in the Midwest, South, and Southeast, reflecting different combinations of crop expansion, intensification, and land‐based mitigation (Figures S1–S3).

Regional differentiation is also pronounced in the energy system. Under Energy‐2040, electricity generation expands most strongly in the Northeast, South and Southeast (Figures S9–S13). By 2050, electricity generation reaches 835 TWh in the Northeast, compared with 489 TWh in Net‐Zero 2050, and 371 TWh in the South, compared with 252 TWh in Net‐Zero 2050. The Northeast expansion is dominated by wind generation, while the composition of growth differs across the other macro‐regions. The Energy‐2040 pathway also produces a strongly differentiated regional biofuel system: by 2050, biofuel production reaches 8.03 EJ in the Southeast, 1.57 EJ in the Midwest and 1.20 EJ in the South, compared with 2.00, 0.27 and 0.33 EJ, respectively, in Net‐Zero 2050 (Figures S14–S18). Regional feedstock requirements consequently diverge as well (Figures S19–S23). These results demonstrate that the national net‐zero pathways do not imply spatially uniform transformations, but instead require region‐specific combinations of land management, electricity infrastructure and bioenergy deployment.

## 4 Discussion

Our results demonstrate that Brazil can advance its net‐zero target by a decade through two contrasting pathways. Importantly, this analysis must be situated within the broader political and diplomatic context. Brazil's current NDC already commits the country to net‐zero GHG emissions by 2050, implying net‐zero CO_2_ until 2040, a target that is among the most ambitious in the Global South. Moreover, both pathways build on existing national commitments and policies, including the target of eliminating illegal deforestation and restoring 12 million hectares of degraded land and forests under the Paris Agreement. Brazil also has the ABC Plan, which directs research, financing, and public resources toward more sustainable agricultural practices.

The growing divergence among the pathways after 2030 is particularly relevant for near‐term policymaking. Although the scenario design does not isolate the effect of advancing the net‐zero year from that of the sector‐specific assumptions, the results show that achieving neutrality by 2040 requires decisions and investments well before 2035. Under AFOLU‐2040, the most consequential near‐term actions involve strengthening deforestation control, expanding restoration supply chains and accelerating the adoption of integrated agricultural systems, widely recognized directions in Brazilian policy, such as the regional plans for the prevention and control of deforestation and in the Native Vegetation Protection Law (Law 12,651/2012). Under Energy‐2040, the critical actions include renewable‐energy deployment, transport electrification, advanced biofuel production, industrial fuel substitution and the early development of CCS infrastructure. Accelerated neutrality would therefore require not only a more stringent final emissions constraint but also an earlier reorientation of policies, investments and institutional capacities. For this sector, Brazil has already established a broader policy framework for climate action through its Climate Plan, which sets sectoral mitigation strategies and provides a framework for aligning national policies with its long‐term climate objectives. In the energy sector, the National Energy Transition Policy complements this framework by establishing a long‐term pathway for the transition of the energy system, while allowing for periodic reviews of its targets and challenges.

Our results reinforce the broad conclusion of previous Brazilian mitigation studies that neither land‐based measures nor energy‐system transformation can be considered in isolation. The prominent role of deforestation control, restoration and low‐carbon agricultural systems in AFOLU‐2040 is consistent with Soterroni et al. (Soterroni et al. 2023), who found that nature‐based solutions are critical for bringing Brazil towards net zero. Similarly, the expansion of transport electrification, advanced biofuels, renewable electricity and CCS in Energy‐2040 is consistent with the technological portfolios identified by Köberle et al. (Köberle et al. 2020) and Poggio et al. (Poggio et al. 2024). The present analysis extends this literature by imposing an explicit net‐zero GHG constraint in 2040 and by comparing two contrasting sectoral strategies under the same socioeconomic assumptions and NDC constraints. It therefore reveals not only the long‐term technological composition of mitigation, but also how the timing and sectoral allocation of action differ during 2025–2035.

In AFOLU‐2040, deforestation nearly halts in the early 2030s, and approximately 20 Mha of degraded lands are restored to native vegetation by 2040, alongside the expansion of agroforestry and integrated crop‐livestock‐forestry (ICLF) systems. In the context of the economic chains of native area restoration and reforestation, this is considered a major advance. Still, it creates significant challenges in meeting these restoration and reforestation volumes due to the need for substantial site expansion to produce native seedlings and seeds, as well as logistical complications between input production sites, the locations where these native‐area restoration and reforestation systems are implemented, and the complex sociocultural aspects involved in the restoration chain. On the other hand, investment in restoration infrastructure can serve as an alternative source of activity and income for local populations by encouraging community‐based restoration and the commercialization of restoration products, thereby linking these activities to programs that stimulate the bioeconomy (Nascimento et al. 2026). Also, this generates substantial negative emissions early in the timeline, positioning land‐use change as the backbone of accelerated neutrality.

In Energy‐2040, the land sector still contributes, but through a different logic: approximately 20 Mha of degraded pasture are converted to more productive systems by 2040, whereas forest restoration plays a smaller role. The Net‐Zero 2050 baseline postpones both restoration and intensification, leaving large areas of degraded land until after 2045. Together, these results highlight the central trade‐off: neutrality can be advanced either by mobilising land‐use transformation at unprecedented speed or by restructuring the energy system to reduce reliance on land‐based removals.

Carbon‐removal portfolios (Figure 5) further illustrate this divergence. AFOLU‐2040 relies heavily on avoided deforestation and reforestation, with agriculture and forestry accounting for more than half of the cumulative reductions by 2040. By contrast, Energy‐2040 shifts the burden of mitigation to energy, industry, and transport, reflecting deep declines in fossil fuel demand, large‐scale deployment of CCS, and increased bioenergy productivity. This scenario requires a contraction of oil and gas production, with refinery closures and declining exports. At the same time, AFOLU‐2040 allows fossil activity to continue at current levels or even expand in the near term. It is also crucial to consider that the transitions foreseen in the ENERGY 2040 scenario, for example, with the intensification of the bioenergy sector, do not represent further conversion of forest to other uses and compromise conservation and restoration commitments. For this reason, it is essential to consider that, actually, in Brazil there are more than 11 million hectares of areas with a legal obligation for restoration (OCF 2026) and Brazil has approximately 75.7 million hectares of pastureland with an intermediate level of degradation and around 39.7 million hectares with a severe level of degradation (LAPIG 2022; dos Santos et al. 2022), that could be restored or converted to agroforestry systems.

The implications for Brazil's energy mix are shown in Figure 6. AFOLU‐2040 maintains oil and gas production at near‐present levels, with fossil fuel demand offset by negative emissions from the land sector. Energy‐2040, in contrast, accelerates the decline of oil use, fosters rapid growth in bioenergy, renewables, and CCS, and fundamentally reshapes the national energy system. These contrasting trajectories highlight the depth of structural transformation required in each scenario, whether in land‐use governance or in energy and industrial systems.

Taken together, these figures underscore that Brazil's net‐zero strategy is not only about timing but also about where the transformation occurs. AFOLU‐2040 prioritises land‐based mitigation, thereby creating space for continued fossil fuel activity, whereas Energy‐2040 requires the energy sector to decarbonise rapidly. Both pathways are technically feasible, but each entails distinctive risks, opportunities, and political trade‐offs.

A strategy combining near‐term AFOLU mitigation with sustained energy‐system transformation could reduce dependence on any single mitigation channel. However, such a combined pathway was not explicitly modeled and should therefore be understood as a policy implication rather than as a direct model result.

The comparatively slower transport electrification in AFOLU‐2040 is an important limitation of this stylised pathway. Although electricity use in transport increases approximately eighteen‐fold between 2025 and 2040, it accounts for only 7.3% of aggregate transport final‐energy consumption in 2040, compared with 31.1% in Energy‐2040. Within passenger transport, the corresponding share in AFOLU‐2040 is approximately 8%. These final‐energy indicators are not directly comparable with electric‐vehicle sales shares because vehicle sales, vehicle stocks and energy consumption have different denominators and turnover dynamics. Nevertheless, electric cars already accounted for 9% of new car sales in Brazil in 2025, and global mitigation pathways identify electrification using low‐emission electricity as a central strategy for reducing transport emissions (IEA International Energy Agency 2023; IPCC 2022b; IEA International Energy Agency 2026). Continued technology diffusion could therefore produce faster electrification than represented in AFOLU‐2040. This scenario should consequently be interpreted as a national land‐led mitigation stress test rather than as a forecast of technological development in a globally interconnected economy (IEA International Energy Agency 2023; IPCC 2022b; IEA International Energy Agency 2026).

The reliance on land‐based mitigation in AFOLU‐2040 has profound governance implications. While Brazil has previously demonstrated its capacity to reduce deforestation rapidly, the reversal of gains in the 2010s highlights the fragility of enforcement (Assis et al. 2019). Achieving neutrality through AFOLU‐2040 would require permanent, uncompromising deforestation controls, unprecedented investment in the restoration industry's production chain, and long‐term monitoring to ensure the permanence of carbon stocks. Any weakening of institutions would undermine neutrality while fossil emissions persist, jeopardising Brazil's international credibility. By contrast, Energy‐2040 reduces dependence on land‐based removals but exposes Brazil to technological and financial risks. Large‐scale deployment of renewables, electrification, and CCS requires massive investment, stable regulatory frameworks, and integration into global supply chains. These risks, while substantial, resemble those faced by other emerging economies undergoing industrial decarbonization.

The regional decomposition further shows that national feasibility does not imply a spatially uniform transition. Land‐based mitigation, renewable‐electricity expansion and biofuel production are distributed differently across Brazil's macro‐regions, reflecting their distinct land resources, energy systems and technology portfolios (Figures S1–S23). This spatial differentiation has important implications for regional planning, including the location of electricity infrastructure, bioenergy supply chains, restoration activities, and agricultural transformation. For example, the Midwest accounts for nearly half of national grain production and contains a substantial share of the agricultural land and land‐based transformations represented by BLUES, whereas the Southeast accounts for the largest share of national electricity consumption. Consequently, translating these technically feasible pathways into practice will depend not only on the availability and deployment of technologies but also on the capacity of state‐level institutions and regional planning mechanisms to respond to differentiated demands and constraints. An accelerated transition will therefore require coordination across federal, state, and municipal levels and sustained engagement among governments, economic sectors, and other stakeholders, particularly where national mitigation objectives intersect with regional development priorities.

However, the regional deployment of technologies should not be interpreted as a direct measure of regional socioeconomic impacts. Employment, income distribution, fiscal revenues, household welfare, and trade competitiveness are not endogenous to BLUES and cannot be inferred directly from technology deployment. Quantifying these consequences would require linking the regional BLUES outputs to macroeconomic, labour‐market, and fiscal models.

Within the scope of the present study, the principal contrast concerns governance and technological risk: AFOLU‐2040 depends on permanent deforestation control and the permanence of land‐based removals (Assis et al. 2019; Bustamante et al. 2019; da Silva et al. 2025; Simonson et al. 2021), whereas Energy‐2040 depends on the rapid deployment of infrastructure, capital‐intensive technologies, and energy‐system infrastructure (La Rovere et al. 2018; Köberle et al. 2020; Poggio et al. 2024; IEA International Energy Agency 2023; IPCC 2022b).

At the global level, advancing Brazil's neutrality target could strengthen the country's contribution to collective climate mitigation. Relative to Net‐Zero 2050, cumulative territorial GHG emissions within Brazil are 1.17 GtCO_2_e lower in Energy‐2040 and 1.45 GtCO_2_e lower in AFOLU‐2040 over the modelled period. These reductions would contribute to global mitigation if they were not offset by higher emissions elsewhere. However, because BLUES represents Brazil as a national system, the model cannot determine whether these reductions would lower global mitigation costs, alter emissions in other regions, or change the probability of limiting warming to 1.5°C. The results nevertheless demonstrate Brazil's potential to undertake earlier mitigation and inform other emerging economies facing trade‐offs between land‐use governance and energy‐system transformation. Diplomatically, Brazil's choice of pathway would also affect its international positioning: AFOLU‐2040 may attract criticism for allowing continued fossil‐fuel activity, whereas Energy‐2040 would align more closely with international energy‐transition pathways.

## 5 Final Remarks

Within the assumptions and national system boundaries represented in BLUES, Brazil could achieve GHG neutrality in 2040 rather than 2050 through alternative mitigation portfolios that place greater emphasis on either land‐sector or energy‐system transformation. AFOLU‐2040 relies on rapid deforestation control, ecosystem restoration, and the expansion of integrated agricultural systems, whereas Energy‐2040 requires earlier transport electrification, renewable‐electricity expansion, advanced biofuels, industrial fuel substitution, and CCS deployment. Relative to Net‐Zero 2050, these pathways reduce cumulative territorial GHG emissions by 1.45 and 1.17 GtCO_2_e, respectively.

These scenarios are not forecasts and should not be interpreted as equally plausible representations of future technological and institutional development. AFOLU‐2040 is a stylised stress test of the extent to which land‐sector mitigation can compensate for a slower energy transition. Its implementation in practice would depend strongly on enforcement capacity and the permanence of land‐based removals. Energy‐2040 reduces this dependence but requires faster infrastructure deployment and technological transformation. Global diffusion of electric vehicles and other low‐carbon technologies could also produce a more rapid energy transition than represented in AFOLU‐2040.

The comparison does not isolate the effect of changing the net‐zero year because the accelerated pathways also include sector‐specific assumptions. Nevertheless, it demonstrates that advancing neutrality changes the timing and composition of mitigation well before 2040. The regional decomposition further shows that these transformations would not be spatially uniform, as land‐use change, electricity expansion and biofuel production are distributed differently across Brazil's macro‐regions. Translating a national pathway into practice would therefore require regionally differentiated policies and coordination across federal, state and municipal levels. A robust national strategy would likely combine immediate land‐sector mitigation with sustained energy‐system transformation, although such a combined pathway was not explicitly modelled here.

## Author Contributions

**Sabrina Matos Carlos:** investigation, data curation. **Susian Martins:** investigation. **Mercedes Bustamante:** conceptualization, writing – original draft. **Felipe Lenti:** investigation, data curation. **Núbia Marques:** investigation. **Nathália Nascimento:** conceptualization, writing – original draft, writing – review and editing, supervision. **Carlos A. Nobre:** funding acquisition, conceptualization, project administration, resources. **Luiz Bernardo Baptista:** investigation, methodology, validation, writing – original draft, writing – review and editing, formal analysis. **Melina Sampaio:** investigation. **Roberto Schaeffer:** conceptualization, writing – original draft, writing – review and editing, validation, methodology, supervision. **Gerd Angelkorte:** conceptualization, investigation, methodology, writing – original draft, validation, visualization, writing – review and editing, formal analysis. **Fabio Diuana:** investigation, writing – original draft, writing – review and editing, methodology, validation, formal analysis. **Laura Vanini Polli:** investigation. **Eduardo Delgado Assad:** conceptualization, writing – original draft. **Alice Assad Wassal:** investigation. **Daiane Pereira de Sousa:** investigation.

## Funding

This study is supported by the Sequoia Climate Foundation and Rockefeller Brothers Foundation, European Union (101183367), Brazilian National Council for Scientific and Technological Development (CNPq), Coordenação de Aperfeiçoamento de Pessoal de Nível Superior ‐ Brasil (CAPES)—Finance Code 001.

## Conflicts of Interest

The authors declare no conflicts of interest.

## Supporting information


**Data S1:** Table S1. Common model settings and exogenous scenario‐specific constraints used in the three net‐zero pathways.
**Table S2:** Selected endogenous outcomes under the three net‐zero pathways.
**Figure S1:** Regional agricultural area by crop in the NDC 2050 scenario.
**Figure S2:** Regional agricultural area by crop in the Energy—2040 scenario.
**Figure S3:** Regional agricultural area by crop in the AFOLU—2040 scenario.
**Figure S4:** Land‐cover change relative to 2020 in the Southeast region.
**Figure S5:** Land‐cover change relative to 2020 in the South region.
**Figure S6:** Land‐cover change relative to 2020 in the Midwest region.
**Figure S7:** Land‐cover change relative to 2020 in the Northeast region.
**Figure S8:** Land‐cover change relative to 2020 in the North region.
**Figure S9:** Electricity generation in the Southeast region.
**Figure S10:** Electricity generation in the South region.
**Figure S11:** Electricity generation in the Midwest region.
**Figure S12:** Electricity generation in the Northeast region.
**Figure S13:** Electricity generation in the North region.
**Figure S14:** Biofuel production in the Southeast region.
**Figure S15:** Biofuel production in the South region.
**Figure S16:** Biofuel production in the Midwest region.
**Figure S17:** Biofuel production in the Northeast region.
**Figure S18:** Biofuel production in the North region.
**Figure S19:** Biofuel feedstock use in the Southeast region.
**Figure S20:** Biofuel feedstock use in the South region.
**Figure S21:** Biofuel feedstock use in the Midwest region.
**Figure S22:** Biofuel feedstock use in the Northeast region.
**Figure S23:** Biofuel feedstock use in the North region.

## Data Availability

The data that support the findings of this study are available on request from the corresponding author. The data are not publicly available due to privacy or ethical restrictions.
