# Supplementary material for: Brazil at the Forefront: What Does It Take To Be Net‐Zero by 2040
Source: Glob Chang Biol. 2026 Sep 15;32(9):e71090. doi: 10.1111/gcb.71090 (PMC13575704; doi:10.1111/gcb.71090)
Supplement: Supplementary file 1 [file GCB-32-e71090-s001.docx]

**Brazil at the forefront: what does it take to be net-zero by 2040**

Gerd Angelkorte^a^, Nathália Nascimento^b,*^, Eduardo Delgado Assad^c^, Luiz Bernardo Baptista^a^, Mercedes Bustamante^d^, Sabrina Matos Carlos^c^, Fabio Diuana^a^, Felipe Lenti^d^, Núbia Marques^d^, Susian Martins^e^, Laura Vanini Polli^c^, Melina Sampaio^b^, Roberto Schaeffer^a^, Daiane Pereira de Sousa^b^, Alice Assad Wassal^e^, Carlos A. Nobre^f^

^a^ Centre for Energy and Environmental Economics (Cenergia), Energy Planning Program (PPE), Coppe, Universidade Federal do Rio de Janeiro, RJ, Brazil

^b^ Departamento de Ciências Florestais, Universidade de São Paulo, SP, Brazil

^c^ Bioeconomy Observatory, Getulio Vargas Foundation FGV/GVagro, São Paulo, Brazil

^d^ Department of Ecology, Institute of Biology, University of Brasília, Brasília, Brazil

^e^ Fauna Projetos Corporation, Campinas, São Paulo, Brazil

^f^ Institute of Advanced Studies, University of São Paulo, Butantã 05508-050, Brazil

^*^Corresponding author: Nathália Nascimento

Email: [nnascimento@usp.br](mailto:nnascimento@usp.br)

**Supplementary Material**

**Table SM 1 | Common model settings and exogenous scenario-specific constraints used in the three net-zero pathways**

| **Scenario dimension** | **Net-Zero 2050** | **AFOLU-2040** | **Energy-2040** |
| --- | --- | --- | --- |
| **Calibration and optimisation period** | Calibrated to 2020; 2025 is the first modelled optimisation period; five-year time steps through 2060 | Same as Net-Zero 2050 | Same as Net-Zero 2050 |
| **Socioeconomic and sectoral demands** | Common population, economic activity, food demand, transport-service demand and other sectoral-demand assumptions | Same as Net-Zero 2050 | Same as Net-Zero 2050 |
| **Techno-economic assumptions** | Common technology costs, efficiencies, lifetimes, resource potentials and fuel-price assumptions | Same as Net-Zero 2050, except where explicitly indicated below | Same as Net-Zero 2050, except where explicitly indicated below |
| **2030 NDC constraint** | Net national GHG emissions of 1,200 MtCO₂e.yr⁻¹ | Same constraint; overachievement allowed | Same constraint; overachievement allowed |
| **2035 NDC constraint** | Net national GHG emissions of 850 MtCO₂e.yr⁻¹ | Same constraint; overachievement allowed | Same constraint; overachievement allowed |
| **Net-zero GHG constraint** | Net-zero national GHG emissions in 2050 | Net-zero national GHG emissions in 2040 | Net-zero national GHG emissions in 2040 |
| **Main scenario design** | Sector-neutral least-cost pathway consistent with the 2050 neutrality constraint | Stylised AFOLU-led pathway combining the 2040 constraint with stronger land-sector assumptions | Stylised energy-led pathway combining the 2040 constraint with stronger energy-system assumptions |
| **Deforestation** | No additional scenario-specific constraint beyond the common policy assumptions | Stronger near-term constraint on native-vegetation loss, including elimination of legal and illegal deforestation by 2030 | No additional AFOLU-specific constraint beyond the common policy assumptions |
| **Restoration and reforestation** | Existing national commitments represented, including the Planaveg benchmark of 12 Mha by 2030 | Restoration constraint strengthened beyond the Planaveg benchmark; Planaveg restoration capacity is allowed to expand endogenously beyond 2030, without the upper bound applied in the other pathways | Restoration limited to a maximum of 50% of the Planaveg benchmark, corresponding to up to 6 Mha by 2030 |
| **Low-carbon agriculture** | Adoption consistent with the common assumptions and ABC+ objectives | Earlier and/or greater adoption than under the ABC+ targets; additional deployment selected endogenously | Implementation of ABC+ agricultural and livestock targets delayed by ten years |
| **Integrated agricultural systems** | Deployment of ICL, ICLFc and ICLFn selected endogenously under common technology constraints | Greater or earlier availability/adoption of integrated systems; additional deployment selected endogenously | Agricultural intensification selected endogenously; no AFOLU-specific acceleration unless otherwise imposed |
| **Transport electrification and modal shifts** | Endogenous deployment under the default technology-availability and adoption constraints | No additional scenario-specific acceleration | Technology-adoption or deployment constraints relaxed to permit faster electrification and modal shifts |
| **Oil consumption and refining** | Determined endogenously under the 2050 emissions constraint | Oil consumption is determined endogenously subject to the 2040 net-zero constraint and expanded industrial/transport mitigation options, with the imposition that the utilisation rate of Brazilian refineries could not fall below 70% | No additional scenario-specific restriction on oil demand |
| **Renewable electricity** | Expansion determined endogenously | Expansion determined endogenously | Expansion determined endogenously |
| **Advanced biofuels** | Deployment determined endogenously | Deployment determined endogenously | Deployment determined endogenously |
| **CCS and BECCS** | Deployment determined endogenously under the common technology and storage assumptions | No additional scenario-specific availability constraint | Greater availability of CCS and BECCS technologies and storage options |
| **Industrial decarbonisation** | Determined endogenously under the 2050 constraint | No additional scenario-specific acceleration | Earlier or greater availability of industrial fuel-substitution and CCS technologies |

Note: NDC, nationally determined contribution; GHG, greenhouse gas; AFOLU, agriculture, forestry and other land use; Planaveg, National Plan for Native Vegetation Recovery; ABC+, Brazilian Plan for Adaptation and Low-Carbon Emissions in Agriculture; ICL, integrated crop-livestock system; ICLFc, integrated crop-livestock-forestry system with commercial forestry; ICLFn, integrated crop-livestock-forestry system with native forest; CCS, carbon capture and storage; BECCS, bioenergy with carbon capture and storage. The table reports only common model settings and exogenous scenario constraints. “Endogenous” indicates that the corresponding level is selected by the cost-optimisation model rather than prescribed as a scenario input. Unless otherwise stated, socioeconomic drivers, sectoral demands, resource potentials and techno-economic assumptions are identical across scenarios.

**Table SM 2 | Selected endogenous outcomes under the three net-zero pathways**

| **Modelled indicator** | **Net-Zero 2050** | **AFOLU-2040** | **Energy-2040** |
| --- | --- | --- | --- |
| **Net GHG emissions in 2030 (MtCO₂e.yr⁻¹)** | 1,200 | 1,153 | 1,168 |
| **Net GHG emissions in 2035 (MtCO₂e.yr⁻¹)** | 850 | 627 | 655 |
| **Reduction in cumulative territorial emissions relative to Net-Zero 2050 (GtCO₂e)** | Reference | 1.45 | 1.17 |
| **Total electricity generation in 2040 (TWh)** | 1,003 | 1,202 | 1,353 |
| **Electricity share of transport final-energy consumption in 2040 (%)** | 4.7 | 7.3 | 31.1 |
| **Transport final-energy consumption in 2040 (PJ)** | 4,132 | 3,947 | 2,254 |
| **Passenger transport final-energy consumption in 2040 (PJ)** | 3,083 | 2,862 | 1,267 |
| **Freight transport final-energy consumption in 2040 (PJ)** | 1,050 | 1,085 | 987 |
| **Transport electricity consumption in 2040 (PJ)** | 193 | 287 | 700 |
| **Passenger transport electricity consumption in 2040 (PJ)** | 137 | 226 | 625 |
| **Freight transport electricity consumption in 2040 (PJ)** | 55 | 61 | 76 |
| **Net change in native-vegetation area between 2020 and 2040 (Mha)** | -1.3 | 19.6 | 4.4 |
| **Net change in the area under integrated agricultural systems between 2020 and 2040 (Mha)** | 23.1 | 24.5 | 12.5 |
| **Net change in the area under agroforestry systems between 2020 and 2040 (Mha)** | 2.0 | 8.4 | 3.1 |
| **Fossil-fuel share of primary energy in 2040 (%)** | 49 | 46 | 22 |
| **Fossil-fuel share of electric generation in 2040 (%)** | 5.1 | 2.4 | 2.2 |
| **Total refinery utilisation factor in 2040 (%)** | 77.5 | 76.1 | 2.9 |
| **Refinery utilisation factor in 2040 – fossil feedstocks (%)** | 77.4 | 76.1 | 0.0 |
| **Refinery utilisation factor in 2040 – co-processing feedstocks (%)** | 0.1 | 0.0 | 2.9 |

Note: Refinery utilisation factor represents the ratio between modelled refinery throughput and available refining capacity.

## **Regional decomposition of the mitigation pathways**

To complement the nationally aggregated results presented in the main manuscript, Figures S1–S23 show the regional distribution of key land- and energy-system transformations across Brazil’s five macro-regions. Figures S1–S3 present agricultural land allocation by crop; Figures S4–S8 present land-cover changes; Figures S9–S13 present electricity generation; Figures S14–S18 present biofuel production; and Figures S19–S23 present biofuel feedstock use. These results represent the regional allocation of mitigation measures within nationally constrained pathways and should not be interpreted as independent regional net-zero targets.

*Regional agricultural land allocation*

**Figure SM 1 | Regional agricultural area by crop in the NDC 2050 scenario**

Note: The scale differs by region.

**Figure SM 2 | Regional agricultural area by crop in the Energy – 2040 scenario**

Note: The scale differs by region.

**Figure SM 3 | Regional agricultural area by crop in the AFOLU – 2040 scenario**

Note: The scale differs by region.

*Regional land-cover change*

**Figure SM 4 | Land-cover change relative to 2020 in the Southeast region**

Note: The scale differs by region.

**Figure SM 5 | Land-cover change relative to 2020 in the South region**

Note: The scale differs by region.

**Figure SM 6 | Land-cover change relative to 2020 in the Midwest region**

Note: The scale differs by region.

**Figure SM 7 | Land-cover change relative to 2020 in the Northeast region**

Note: The scale differs by region.

**Figure SM 8 | Land-cover change relative to 2020 in the North region**

Note: The scale differs by region.

*Regional electricity generation*

**Figure SM 9 | Electricity generation in the Southeast region**

Note: The scale differs by region.

**Figure SM 10 | Electricity generation in the South region**

Note: The scale differs by region.

**Figure SM 11 | Electricity generation in the Midwest region**

Note: The scale differs by region.

**Figure SM 12 | Electricity generation in the Northeast region**

Note: The scale differs by region.

**Figure SM 13 | Electricity generation in the North region**

Note: The scale differs by region.

*Regional biofuel production*

**Figure SM 14 | Biofuel production in the Southeast region**

Note: The scale differs by region.

**Figure SM 15 | Biofuel production in the South region**

Note: The scale differs by region.

**Figure SM 16 | Biofuel production in the Midwest region**

Note: The scale differs by region.

**Figure SM 17 | Biofuel production in the Northeast region**

Note: The scale differs by region.

**Figure SM 18 | Biofuel production in the North region**

Note: The scale differs by region.

*Regional biofuel feedstock use*

**Figure SM 19 | Biofuel feedstock use in the Southeast region**

Note: The scale differs by region.

**Figure SM 20 | Biofuel feedstock use in the South region**

Note: The scale differs by region.

**Figure SM 21 | Biofuel feedstock use in the Midwest region**

Note: The scale differs by region.

**Figure SM 22 | Biofuel feedstock use in the Northeast region**

Note: The scale differs by region.

**Figure SM 23 | Biofuel feedstock use in the North region**

Note: The scale differs by region.
